# Implementation of multiomic mass spectrometry approaches for the evaluation of human health following environmental exposure

**DOI:** 10.1039/d3mo00214d

**Published:** 2024-03-26

**Authors:** Christina R. Ferreira, Paulo Clairmont F. de Lima Gomes, Kiley Marie Robison‡, Bruce R. Cooper‡, Jonathan H. Shannahan

**Affiliations:** a Purdue Metabolite Profiling Facility, Purdue University West Lafayette IN 47907 USA cferrei@purdue.edu; b Sao Paulo State University Julio de Mesquita Filho, Institute of Chemistry Araraquara Sao Paulo Brazil; c School of Health Sciences, College of Health and Human Sciences, Purdue University West Lafayette IN 47907 USA

## Abstract

Omics analyses collectively refer to the possibility of profiling genetic variants, RNA, epigenetic markers, proteins, lipids, and metabolites. The most common analytical approaches used for detecting molecules present within biofluids related to metabolism are vibrational spectroscopy techniques, represented by infrared, Raman, and nuclear magnetic resonance (NMR) spectroscopies and mass spectrometry (MS). Omics-based assessments utilizing MS are rapidly expanding and being applied to various scientific disciplines and clinical settings. Most of the omics instruments are operated by specialists in dedicated laboratories; however, the development of miniature portable omics has made the technology more available to users for field applications. Variations in molecular information gained from omics approaches are useful for evaluating human health following environmental exposure and the development and progression of numerous diseases. As MS technology develops so do statistical and machine learning methods for the detection of molecular deviations from personalized metabolism, which are correlated to altered health conditions, and they are intended to provide a multi-disciplinary overview for researchers interested in adding multiomic analysis to their current efforts. This includes an introduction to mass spectrometry-based omics technologies, current state-of-the-art capabilities and their respective strengths and limitations for surveying molecular information. Furthermore, we describe how knowledge gained from these assessments can be applied to personalized medicine and diagnostic strategies.

## Abstract 2

This review introduces advancements in multiomic mass spectrometry which revolutionized our knowledge of complex biological processes across scientific disciplines, exposure scenarios, and diseases, benefiting diagnostic and treatment strategies.

## Graphical abstract

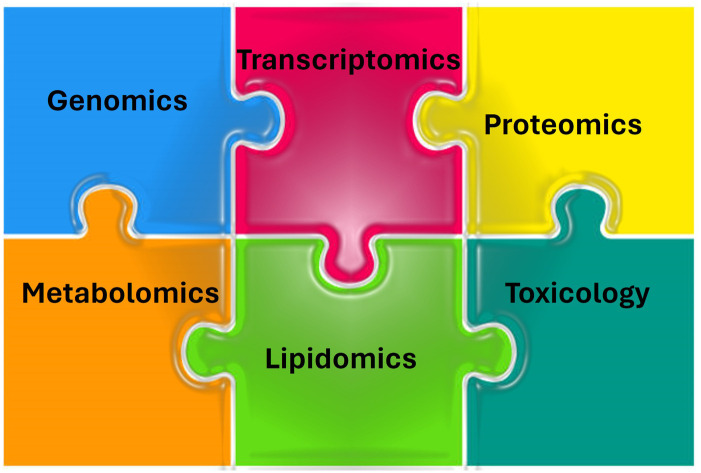

## 1 Introduction

This review is intended to provide a description of sample types and techniques used for multiomics studies. It also features research, instrumentation and perspectives on personalized medicine. The authors’ goal is to showcase the power of multiomic analyses and provide background materials for researchers interested in diving into combining datasets related to the different levels of biological information present in cells, tissues and organisms. Biofluids used for the molecular analysis of metabolism and for diagnostic purposes include blood (as well as serum and plasma), urine, saliva, skin sebum, cerebrospinal fluid, gut aspirate, bile, amniotic fluid, synovial fluid, exhaled breath or breath condensate, nasal secretions, intact tissue, and tissue extracts. For the purpose of this review, we will focus on the usage of readily available and easily accessible biofluids, including blood, serum, plasma, saliva, sweat, skin sebum, and urine, for molecular phenotyping and health monitoring. Even though next generation DNA sequencing allows for the detection of genetic conditions in biofluids, the chemical composition of biofluids can be used as a “real-time” molecular phenotypical baseline due to its dynamic changes. Changes in the metabolite composition of diverse biofluids can potentially be utilized to detect health issues or a variety of exposure-related health consequences.^1,2^ Information from existing omics databases, which contain molecular features associated with disease conditions and toxicity responses, can be used to understand metabolic mechanisms of diseases and interpret deviations from established baselines.^3,4^ Currently, the greatest challenge in employing metabolic phenotyping is that large portions of the human metabolome composition are unknown.^5,6^ The most utilized omics approaches are genomics, transcriptomics, proteomics, and metabolomics. The integration of multiple approaches can provide deeper and more comprehensive insight into complex biological processes. Multi-omics approaches fit well into the concept of precision medicine and mass spectrometry (MS) is the dominating analytical technology for the omics approaches that can monitor metabolic phenotypes, namely proteomics and metabolomics.^7,8^ MS systems can also be applied to the screening of biological reactions, as well as to the establishment of the metabolic transformations of drugs and chemicals. Currently, drug development is largely based on omics approaches and pharmacokinetics studies are the basis of biosafety and efficacy studies. We discuss the portability aspect of MS, which is expected to become the implementation strategy for precision medicine. Lastly, we survey efforts and methods for dealing with data interrelationships among existing omics that are packaged as data analysis workflows and in dedicated software programs.

## 2 Multi-omics approaches

### 2.1 Genomics

The first human genome sequencing was reported in 2003. It was a 13 year-long project and approximately 21 300 genes were detected. Next-generation sequencing (NGS) has only been commercially available for about 12 years. Nonetheless, the meteoric increase in sequencing throughput with NGS has dramatically changed our understanding of our genome and ourselves. NGS has also reduced the cost of generating sequence data and a plethora of sequence-based methods for probing a genome have emerged using NGS as the readout and have been applied to many species. The price of genome sequencing significantly dropped from $100 000 000 to $1000. Gene mutations are not the only root causes identified for a disease. Multiple environmental factors that directly influence the metabolism have been found to play a crucial role in health. NGS methods have also entered the medical realm driven by short-read generation (150 bp), but new platforms have emerged and are now capable of generating long multi-kilobase reads. The latter platforms enable reference-independent genome assemblies and long-range haplotype generation. Rapid DNA and RNA sequencing is a mainstream technology in personalized medicine and will continue to have an increasing impact on biology and medicine.^9^ NGS is currently established as a test method for germline (inherited) and somatic (acquired) genetic mutations in many clinical laboratories. For inherited diseases, testing for germline mutations may include targeted panel, whole exome, whole genome, or mitochondrial DNA sequencing.^10,11^ Targeted panel testing, which varies between laboratories, is possible for a wide variety of inherited disorders such as immune deficiencies, bone marrow failure syndromes, blindness, deafness, mitochondrial disorders, renal disorders, neurologic disorders, connective tissue disorders, cardiomyopathies, and cancer predisposition syndromes, among others.^12–19^ Targeted panels for genes associated with a clinical phenotype are usually the first line of testing for inherited disorders, while whole exome sequencing is reserved for cases in which targeted testing has been uninformative.^20,21^ Targeted panels for cancer testing also vary between laboratories. Targeted panels may be broad, including genes responsible for both solid and hematologic malignancies, or may be more focused for a particular type of malignancy (such as myeloid neoplasms).^22^ Any given gene within a panel may be completely sequenced or only partially sequenced (*e.g.*, hotspot regions). For both germline and somatic testing, it is important to know the content of the targeted panels when deciding on using a test. Whole exome and whole genome sequencing are not currently used clinically for oncology testing. Several new applications for NGS have more recently moved into the clinical arena or are being actively researched for clinical use, including circulating tumor DNA testing, human leukocyte antigen (HLA) typing, microbial analysis, RNA sequencing and expression, and methylation. Some of these new uses of NGS may be facilitated by the unique advantages of new instruments.

### 2.2 Transcriptomics

In the last few decades, transcriptome profiling has been one of the most utilized approaches to investigate human diseases at the molecular level. Molecular biomarkers and therapeutic targets have been found for several human pathologies through the quantification of gene expression levels and allele-specific expression. Large scale transcriptomics can be performed in a single experiment and can be used to identify novel genes, splice isoforms, and fusion transcripts and to investigate the world of non-coding RNAs at an unprecedented level. RNA sequencing has also been employed in important projects, like ENCODE (Encyclopedia of DNA Elements) and TCGA (The Cancer Genome Atlas), to provide a snapshot of the transcriptome of dozens of cell lines and thousands of primary tumor specimens. Moreover, transcriptomics studies have also paved the way for the development of data integration approaches.^23^ However, like any other experimental approach, transcriptomics has its limitations: it is an inappropriate method to identify genes with large impacts on adaptive responses to the environment because: (i) genes with large impacts on fitness are rare; (ii) a large change in gene expression does not necessarily equate to a large effect on fitness; and (iii) protein activity is most relevant to fitness, and mRNA abundance is an unreliable indicator of protein activity.^24^

### 2.3 Proteomics

Proteomics is the study of the interactions, function, composition, and structures of proteins and their cellular activities.^25^ Proteomics provides a better understanding of the structure and function of the organism than genomics. However, it is much more complicated than genomics because the protein expression is altered according to time and environmental conditions.^26^ It is estimated that there are almost one million human proteins, many of which contain some modifications such as post-translational modifications (PTMs). However, it is also estimated that the human genome codes for about 26 000–31 000 proteins.^27^ There are a variety of proteomics techniques including one-dimensional (1D) and two-dimensional (2D) gel electrophoresis (2-DE), as well as gel-free high-throughput screening technologies such as multidimensional protein identification technology, stable isotope labeling with amino acids in cell culture, isotope-coded affinity tag, and isobaric tagging for relative and absolute quantitation. Shotgun proteomics, 2D difference gel electrophoresis (2D-DIGE), and protein microarrays can be used to investigate tissues, organelles, and cells. Large-scale western blot assays, multiple reaction monitoring assays, and label-free quantification of high mass resolution liquid chromatography (LC)-tandem mass spectrometry (MS) are commonly used for high-throughput processing.^28^ Limitations related to the use of proteomics for diagnosis are related to the fact the disease-related proteins are often present at low concentrations mixed with various other proteins of much higher abundance, which makes it more difficult to identify them. Another common drawback is nonspecific adsorption of non-target proteins onto the surface of biosensors. Enzyme-linked immunosorbent assay (ELISA) is commonly used for the detection of specific proteins in biofluids and typically employs antibodies that are raised in animals directed against specific biomarkers. Other technologies used to detect specific proteins are electrochemical immunoassays, surface enhanced Raman spectroscopy (SERS), flow cytometry and protein microarrays.^29^ The main limitation of portable systems for specific protein detection is the cost and the difficulty in profiling panels of proteins that can be related to a metabolic baseline.

### 2.4 Metabolomics

The composition of small molecule metabolites or chemicals that can be found in a cell, a tissue, an organism or even an environmental sample (such as in air or sewage) is defined as the metabolome. Metabolomics, which is the study of the metabolome, is one of the most recent branches of the omics sciences. What makes metabolomics so different is that it focuses on small molecules (*i.e.*, chemicals with a molecular weight less than 1500 Daltons), while the other omics fields focus on big molecules (*i.e.*, DNA, RNA, and proteins). Metabolomics became so interesting because metabolites are the downstream products arising from the collective activities of the genome, the transcriptome and the proteome interacting with their environment. In other words, the metabolome is the closest omics to a molecular phenotype.^30^ Applications of metabolomics span a wide range of disciplines including health and various diseases, pharmacology, drug development, toxicology, environment, plants, and food and nutrition. However, most of the studies are focused on improving the mechanistic understanding, along with prevention, early diagnosis, and management of human health and diseases. Metabolism screening is fundamental in interpreting a patient's phenotype. Newborn bloodspot screening (NBS) for phenylketonuria was reported in 1959. In 2012 9.5 million babies were screened for inborn errors of metabolism. Current technology is tandem mass spectrometry due to the short analysis time, high sensitivity, and selectivity to quantify NBS metabolites in dried blood spots of several hundreds of samples per day. In some countries every newborn is screened. Other examples are therapeutic drug monitoring (TDM) for inflammatory bowel diseases (IBD) and synthetic opioids. The most common analytical approaches used for metabolomics are vibrational spectroscopy techniques, represented by infrared, Raman, and nuclear magnetic resonance (NMR) spectroscopies and mass spectrometry (MS). Among these, NMR spectroscopy and MS are the two most commonly employed methods in the metabolomics field. MS is a highly sensitive method, and it enables the analysis of several hundreds to thousands of metabolites from a single measurement and on a routine basis. In MS analysis, often, metabolites can be directly analyzed using ambient ionization or can be subjected to chromatographic separation using liquid chromatography, gas chromatography, or capillary electrophoresis prior to detection. A variety of MS methods are often used for analysis of different classes of metabolites from the same samples to achieve a wider coverage of the metabolome. NMR spectroscopy is often used without combining with any sample preprocessing or separation techniques and provides data complementary to MS, mostly due to sensitivity issues. Peaks in the NMR spectra can be reliably assigned to specific metabolites especially from pure compounds and peak intensities are directly proportional to the number of contributing nuclei.^31^ Vibrational spectroscopy (infrared and Raman spectroscopies) offers rapid, high-throughput, and non-destructive analysis of a wide range of samples through chemical “fingerprinting”. The basis of vibrational spectroscopy is the transitions between quantized vibrational energy states of molecules due to the interaction between the material and the radiation from a light source.^32,33^ Vibrational spectroscopy includes infrared and Raman spectroscopies. Even though both the near-IR (12 500–4000 cm^−1^) and mid-IR (4000–400 cm^−1^) are part of the infrared spectrum, most medical researchers focus on the mid-IR part of the spectrum because the fundamental vibrations in the mid-IR region provide sharper bands and more information on disease diagnosis rather than the overtone and harmonic vibrations that are provided by the near-IR region.^34,35^ Mid-IR spectroscopy is based on the interaction between the sample and the IR beam, which is absorbed by the functional groups in the sample that vibrate in stretching, bending, deformation modes or their combination, and provides the fingerprint characteristics of the chemical or biochemical substances in the sample.^34^ A major hurdle for FT-IR spectroscopy is the interference of the water in the mid-IR region, which masks some key biochemical information, especially in the amide I (1650 cm^−1^) and lipid (3000–3500 cm^−1^) absorption regions, and the water absorption could inhibit the light from penetrating the sample.^36,37^ There are several approaches to overcome the water problem including the removal of the pure or scaled water spectrum from the acquired spectrum, dehydrating the sample, using D_2_O solution, or lowering the effective path length significantly by using the attenuated total reflectance (ATR) as a sampling technique.^34,36,37^ Raman spectroscopy is an inelastic light-scattering phenomenon; the incident photon is irradiated on the sample, and the molecules scatter the light. Although most of the scattered light has the same frequency as the incident light, some of them have different frequencies due to the interaction between the oscillation of light and molecular vibration. This phenomenon is called Raman scattering and, unlike IR spectroscopy, Raman spectroscopy has a very weak water signal and minimal water interference, which is an advantage for the analysis of the biological samples.^38^ Raman spectroscopy can offer direct measurements of biofluids and single cells and *in vitro* or even *in vivo* fiber-optic sampling of bladder and prostate, esophagus, skin, cervix, and arteries.^34^ Furthermore, Raman spectroscopy is a non-destructive and non-invasive (wavelength and power-dependent) technique; it requires minimal sample preparation and simultaneous detection of macromolecules suitable for chemical analysis, quantification, classification, and the imaging of biological samples.^39^ On the other hand, the Raman effect is very weak, and only 1 in 108 photons undergo Raman scattering;^40^ to overcome this drawback longer acquisition times could be used, which could cause damage to the sample due to the laser exposure.^39^ The other method to amplify the inherent signal weakness of Raman spectroscopy is by using the surface-enhanced Raman scattering (SERS) technique. SERS uses nanoscale roughened metallic surfaces (typically gold or silver), which could greatly enhance the order of the Raman signal (108).^41^ The signal enhancement can increase even up to 1011 with surface-enhanced resonance Raman spectroscopy (SERRS). Advancements in the Raman instrumentation along with the SERS phenomenon have boosted the application of Raman as a diagnosis tool.^42^ The other hurdle for Raman spectroscopy is the fluorescence interference, which happens when visible wavelength lasers are used. Especially during *in vivo* analysis, the fluorescence background signal can dominate the fingerprint region of the spectra.^34,39^ The fluorescence interference can be removed mathematically or by illuminating the sample with the laser beam for a long time as a pre-treatment (this process is also known as “bleaching” or “photobleaching”) or by using longer wavelength lasers (*i.e.*, 1064 nm).^34^

### 2.5 Lipidomics

The same analytical approaches used for metabolomics are also applied to the study of the molecular composition of lipids. Lipidomics is considered a sub-area of metabolomics. Lipids have a variety of cellular functions (fuel for cell growth, signaling molecules, stimulatory agents, and can have an inhibitory effect on enzymes). From a chemical point of view, lipids are a heterogeneous pool of compounds that contain either fatty acyl/alkyl, sphingosine, or isoprene moieties as their hydrophobic building blocks. In 2005, lipids were classified into eight categories: fatty acyls, glycerolipids, glycerophospholipids, sphingolipids, sterols, prenol lipids, saccharolipids, and polyketides.^43^ Since lipids play a crucial role in many biological processes, any imbalance in their homeostasis can lead to serious conditions in living organisms, such as chronic inflammation, cardiovascular diseases, diabetes, and neurodegenerative diseases, to name just a few. The method of choice for the analysis of lipid molecules or their huge assemblies (known as the lipidome) is undoubtedly mass spectrometry, due to its sensitivity and specificity.^44^ Because of the inherent chemical complexity of the lipidome and the consequent challenges associated with analyzing it, progress in the field of lipidomics lagged behind the progress made in other omics disciplines.^45^ Even though chromatographic separation followed by high resolution mass spectrometry is commonly used for lipidomics studies, ‘shotgun lipidomics’, involving direct injection and infusion (*i.e.*, no sample separation), are informative, quantitative, and fast.^46–48^ In recent years, desorption electrospray ionization (DESI) has become a noteworthy option for direct infusion lipidomics. In a comparative study with LC-MS, it was shown that DESI-MS forms different adducts than LC-MS, but when adjusted for these different adducts, the mass spectra show a very high degree of correlation in the determined lipid composition.^49^ The major advantage of DESI in lipidomics is the ability to use it in combination with a gas-phase technology named ion mobility.^50^

### 2.6 Exposomics

**Fig. 1:**
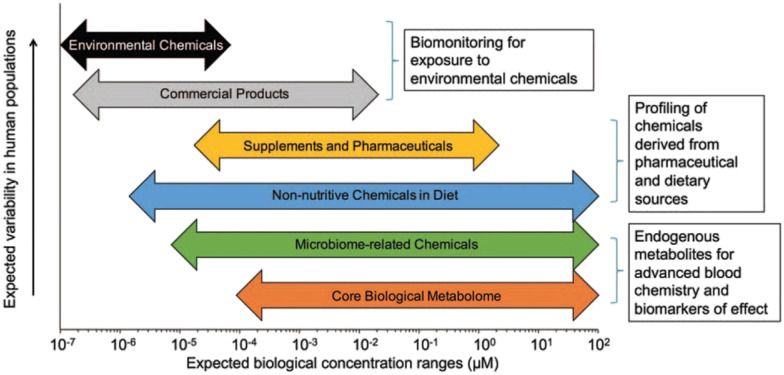
Analytical sensitivity is important for measurement of endogenous metabolites, especially environmental chemicals, which are often present at four or five orders-of-magnitude lower abundance compared to endogenous metabolites. Published by Douglas *et al.*^54^ after adaptation from Walker *et al.*^67^ Adapted with copyright permission from Wolters Kluwer Health, Inc.

Health effects of a chemical depend on numerous factors beyond dosage. The concept of exposome was introduced in 2005 to study the health effects of cumulative environmental exposures and concomitant biological responses from conception until death.^51–53^ Derived from the term exposure, the exposome is an omic-scale characterization of the nongenetic drivers of health and disease. The exposome represents a shift toward comprehensive exposure assessment by assessing multiple, co-occurring exposures that may be found at low concentrations, like real-life exposure conditions. It also promotes the understanding about the interactions of exposures with endogenous processes influencing their biological effects and enables the identification of critical windows of exposure over the life course. Nonetheless, the study of exposome is challenging due to the low levels of compounds of interest (Fig. 1). Because endo- and exogenous chemicals are simultaneously detected, metabolomics provides an integrated measurement to link exposure to internal dose, biological response, and disease pathobiology.^54–56^ By not limiting detected analytes to those selected a priori, untargeted metabolomics greatly expands surveillance of environmental chemicals, detection of new xenobiotic metabolites, and identification of previously uncharacterized pollutants.^57–61^ Curation of metabolomics data to provide confirmed identification of the chemicals associated with the mass spectral features represents a critical research need. Despite this limitation, the unbiased and global characterization of metabolic responses enables the generation of new hypotheses for delineating toxicological mechanisms underlying chemical exposure in model systems, as reviewed by Niedzwiecki *et al.*, 2019.^62^ In humans, proteomic studies have identified immune- and inflammation-related proteins associated with exposure to diesel exhaust^63^ and polycyclic aromatic hydrocarbons (PAHs).^64^ Continued development of multiplexed proteomics has considerable potential for characterizing biological responses, though traditional untargeted proteomics is challenging due to the difficulties in detecting low-abundance proteins in serum. Epigenomics is a key approach to evaluate exposure history and allostatic load.^65,66^ In human cells, methylation of DNA occurs at the CpG dinucleotides in the cytosine C5 position. While tens of millions of CpG sites are present within the human genome, current high-throughput assays based on massively parallel sequencing of DNA with bisulfite conversions provide measures of up to 850 000 CpG sites. Epigenome-wide association studies have found distinct methylation patterns associated with chemical exposure, providing insight into mechanisms underlying biological responses and diseases.^62^

### 2.7 Microbiome

The gut microbiota is very diverse and contains many culturable and unculturable members that play critical roles in host health and disease. The members of the gut microbiota include archaea, bacteria, viruses, and fungi^68^ and these organisms interact with each other and with the host. Metagenomic sequencing techniques have made it possible to study the microbial communities in the gut under different conditions and they help to detect alterations that occur during disease conditions. These techniques have been helpful in distinguishing healthy subjects from cancer,^69^ inflammatory bowel disease,^70^ and autism^71^ patients. However, the presence of a microbe does not give any indication of its role in the gut. Also, the metabolic potentials of uncultured microbes are unknown, and this makes metagenomics data alone inadequate in providing information about the gut microbial ecology.^72^ Meanwhile, as only the DNA of live and active microbes is transcribed into RNA, analyzing gut microbial mRNA (metatranscriptomics) has become a robust technique for detecting and quantifying transcribed mRNAs to predict their metabolic potential.^73^ However, since not all mRNAs are translated into proteins, metaproteomics, an analytical technique that can analyze gut microbial proteins in samples, is usually used to detect and quantify such proteins.^74^ Other microbial metabolites such as lipids, carbohydrates, and some other biomolecules have also been shown to be essential for microbe–host interaction.^75^ For this reason, multi-omics approaches are increasingly being applied to identify gut microbial metabolites and host–microbe cometabolites, which may help unravelling the complex interaction between host and gut microbes.^75,76^

## 3 Examples of omics and multiomics studies focused on health and disease

### 3.1 Baseline *vs.* follow-up monitoring

**Fig. 2:**
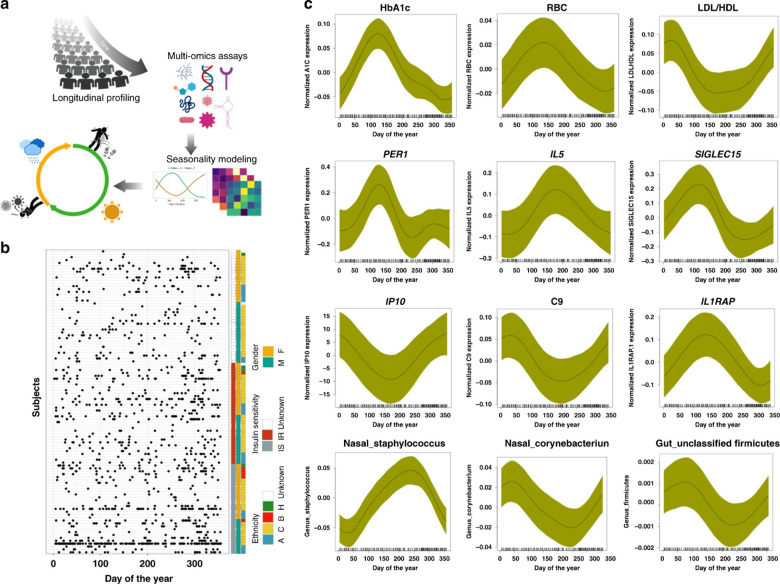
Schematic view of a multi-omics effort named integrative personal omics profiling (iPOP) to study seasonal influences on the human body. (a) The omics assays included immune molecule profiling, proteomics, metabolomics, transcriptomics, and microbial profiling (gut and nasal), in conjunction with clinical lab tests and meteorological measurements. (b) Subjects and sampling timepoints for everyone, as well as ethnicity (A: Asian, B: black, C: Caucasian), insulin sensitivity (IS) and insulin resistance (IR), and gender information (M: male, F: female). (c) Examples of omics analytes with seasonal patterns (transcripts, cytokines, metabolites, proteins, clinical lab tests, gut, and nasal microbiome). The *x*-axis shows the days of the year (1–365 days) and *y*-axis shows the normalized expression/abundance values. The samples were collected up to 4 years and aggregated and mapped to 1-year-long time frame. The shaded area represents 95% confidence bounds computed as ±1.96 standard deviation of model coefficients.^54^ Adapted with copyright permission from Springer Nature Publishing.

The iPOP (integrated Personal Omics Profiling) study is an example of a current effort to establish a phenotypical baseline and use it for health status evaluation. The effort includes a longitudinal study of approximately 100 individuals meant to help lay a foundation for precision personalized medicine through the unprecedented deep biochemical profiling of generally healthy individuals and understanding environmental conditions, such as seasonal influences (Fig. 2). It is designed to understand what “healthy” biochemical and physiological profiles look like at a personal level and what happens when people become ill. The study was designed and performed at Stanford University.^54^

Over the course of several years samples were collected from participants at regular intervals, both while they were in good health and in times of illness or significant stress. Whole genome sequencing was performed on all participants, and other omics data collected include information on how the genome is expressed (transcriptome, proteome, methylome), bacteria and other microorganisms in the gut and on the skin (microbiome), and the intermediate products of metabolism (metabolome). Data are also collected on participants' diets, stress levels, activity levels, and personal and family medical history. Wearable devices enable tracking of participants physiology and activity. Altogether, billions of measurements were made every time someone was sampled.

**Fig. 3:**
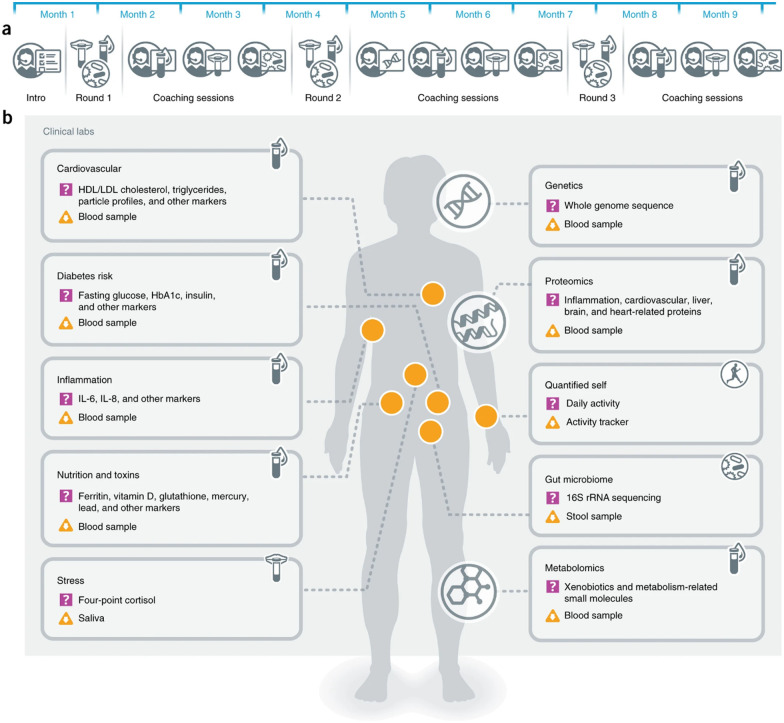
(a) Timeline of important events and (b) schematic of the data collected for the generation and analysis of personal, dense, dynamic data clouds called the Pioneer 100 Wellness Project (P100). Personal data for 108 individuals were collected during a 9-month period, including whole genome sequences; clinical tests, metabolomes, proteomes, and microbiomes at three time points; and daily activity tracking. Using these data, a correlation network that revealed communities of related analytes associated with physiology and disease was generated. Connectivity within analyte communities enabled the identification of known and candidate biomarkers (*e.g.*, gamma-glutamyltyrosine was densely interconnected with clinical analytes for cardiometabolic disease). Polygenic scores from genome-wide association studies (GWAS) for 127 traits and diseases were used to discover molecular correlates of polygenic risk (*e.g.*, genetic risk for inflammatory bowel disease was negatively correlated with plasma cystine).^78^ Adapted with copyright permission from Springer Nature Publishing.

Another effort, named the The Pioneer 100 Wellness Project (P100),^78^ is presented in Fig. 3, which computed thousands of statistically significant inter-omic correlations using personal, dense, dynamic data clouds to identify many associations that could be followed up with perturbation experiments. The correlations were partitioned into data communities to establish biomarkers in context within biological networks. This approach led to the identification of putative biomarkers such as gamma-glutamyltyrosine for cardiometabolic disease. The clinical biomarker of many participants significantly changed regarding the disease background during the study (*e.g.*, type 2 diabetes and cardiovascular risk factors). Together this study indicates that personal, dense, dynamic data clouds embody the essence of precision medicine and present possibilities for the discovery of important medical applications.^78^

### 3.2 Environmental exposure

In the United States, over 85 000 chemicals are registered with the EPA for manufacture, import, and use in commercial products. Additionally, approximately 40 000 pesticide formulations, 100 000 dietary phytochemicals, and 5000 other chemicals are approved for use as inert ingredients. Also 7500 compounds are registered by the US Food and Drug Administration as drugs or food additives. An individual's history of these exposures over a lifetime—that is, their chemical experience—may contribute directly to phenotype and health. In almost all cases, limited information is available about these chemicals in terms of their distributions across populations, the health effects of low-level exposures, and the influence of complex mixtures encountered in real-world scenarios. The adequate characterization of an individual's chemical burden will require the ability to measure upwards of 1 million chemicals routinely across the lifespan in a cost-effective and efficient manner.^79^ Exposure to environmental chemicals can initiate local and global changes in gene transcription, enzyme activity, metabolite pathway alterations, and protein synthesis/folding. As a result, micro- and macroscale interactions occur among these systems that can be characterized to study dose–response relationships. In 2011, the United States Institute of Medicine (IOM) recommended that the Department of Defense (DoD) collect individual breathing zone samples and conduct long-term studies of troop health outcomes to address concerns about perceived health risks resulting from exposure during deployment.^80–82^ Realistically, there are inherent limits to exposure assessment in deployed settings. For example, the use of personal monitoring equipment limits mobility in active combat situations, logistics of sampler collection is challenging with large-scale troop movements, and assessment for biologically relevant dose requires additional molecular measurements. Furthermore, the post-exposure window of opportunity for measuring exposures or immediate consequences may range from hours to days for some agents. Therefore, valid and reliable measures are needed to characterize exposures that do not disrupt effective operation during deployment. Retrospective profiling of biological specimens collected pre- and post-deployment for biomarkers of exposure, effect, and susceptibility provides a means of assessing the occurrence of chemical exposure related to poor health outcomes. Through the DoD Serum Repository (DoDSR), an extensive system exists for collection, cataloguing, and storing of serum samples collected pre- and post-deployment from armed forces personnel.^83,84^ Incorporating chemical screening measures using serum samples collected under the current DoDSR framework could therefore be completed with minimum disruption to military operations.^54^

Gas or liquid chromatography with ultrahigh-accuracy mass spectrometry is the most promising analytical technology for an exposome platform for precision medicine.^54,85–88^ Due to increases in scan speed and data extraction algorithms, modern instruments can detect 20 000–100 000 unique chemical signals in small volumes (<150 μL). Including triplicate injections improves reliability of peak detection when studying exposures that occur in a small subset of the population. Combined with a technique known as reference standardization, MS can determine absolute concentrations of biomarkers for the assessment of potential risks from exposures.^88^ Additionally, MS is cost-effective relative to other biomonitoring platforms.^54^ Further cost reduction is possible through focused analysis of high-abundance metabolites and exposure markers. It reliably detects approximately 1000 common endogenous metabolites, commercial products, and drug metabolites with coefficient of variation (CV) less than 10%.^85,87–89^ By limiting detection to chemical signals with low CVs, reducing runtimes, and employing automation, samples could theoretically be processed with a throughput of 500 samples/day (125 000 samples/instrument-year) at a cost of $5 per sample. Thus, sufficient chemical coverage for the purposes of precision medicine and the detection of environmental exposures and related bioeffects could be obtained at a low cost with available technology.

Transcriptomics, proteomics, metabolomics, and lipidomics data revealed cigarette smoke induced inflammatory and oxidative stress response, as well as lipid/surfactant alterations. However, at matched nicotine concentrations, aerosol exposure from carbon heated tobacco products and tobacco heating systems, these effects were either limited or absent as described by Titz *et al.*^90^

Herron *et al.*^91^ used lipidomics and transcriptomics to demonstrate that benzalkonium chlorides (BACs) can cross the blood–placental barrier and embryonic blood–brain barrier, resulting in altered sterol and lipid homeostasis. When fetuses are exposed to BACs *in utero*, signaling pathways important for neuronal development, such as LXR/RXR and glutamatergic signaling, are negatively affected.

### 3.3 Metabolic diseases

**Fig. 4:**
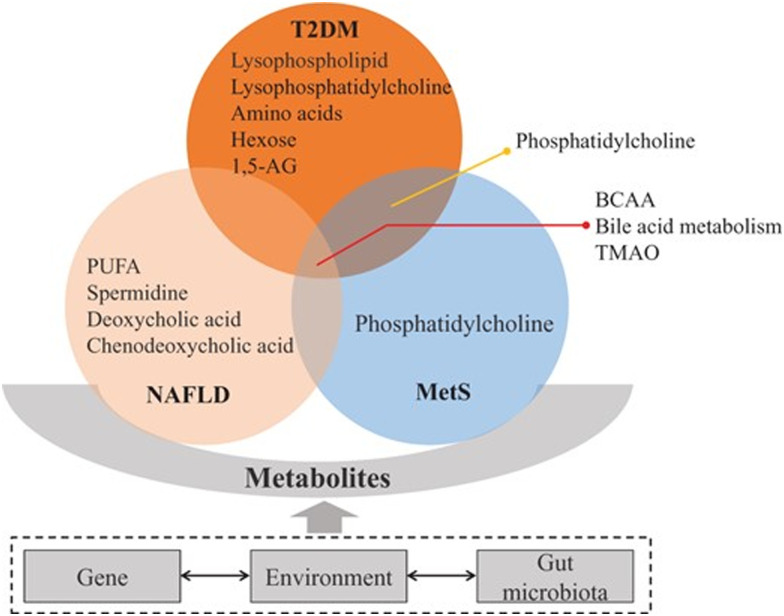
Metabolites tightly connected with type 2 diabetes mellitus (T2DM), non-alcoholic fatty liver disease (NAFLD), and metabolic syndrome (MetS). These conditions are associated with insulin resistance, bile acid and lipid metabolism changes. Among these pathways, branched-chain amino acids (BCAA), bile acid metabolism and trimethylamine *N*-oxide (TMAO) are implicated in T2DM, NAFLD, and MetS. Phosphatidylcholine is associated with T2DM and MetS. Other metabolites have been related specifically to one of these diseases.^92^ Adapted with copyright permission from Oxford University Press.

Metabolic diseases including type 2 diabetes mellitus (T2DM), non-alcoholic fatty liver disease (NAFLD), and metabolic syndrome (MetS) are alarming health burdens around the world and examples of multi-omics conditions since they are multifactorial metabolic disorders based on the interactions between genetics and environment. The multiple components of these diseases have been recently reviewed by Hu and Jia.^92^ Familial aggregation,^93^ ethnic differences,^94^ and higher concordance rate of T2DM in monozygotic than in dizygotic twins^95^ indicate genetic contribution to T2DM. For NAFLD, there have been biochemical, imaging, genetic, and other omics biomarkers for its staging and progression.^96^ A strong heritability of NAFLD susceptibility has been identified in epidemiological, family, and twin studies.^97,98^ Studies on T2DM patients have identified transcriptional differences in islets, liver, muscle, adipose tissue, and peripheral blood using RNA sequencing transcriptomics studies on NAFLD mainly focusing on the liver. A differential expression analysis in severe *vs.* non-severe NAFLD and normal liver^99^ showed 320 genes differentially expressed in severe NAFLD. Also, several studies identified epigenetic changes in T2DM patients, and the regions were also associated with differential expression of genes. High-fat diet (HFD) can induce modifications in the chromatin structure, thereby contributing to metabolic disease.^100^ FAIRE-seq (a method in molecular biology used for determining the sequences of DNA regions in the genome associated with regulatory activity) was performed in the livers of C57BL/6J mice induced by HFD and control diet, which identified 28 484 open chromatin sites in control and 28 253 sites in high-fat livers. There are several proteins associated with incidence and progression of T2DM. The approach of constructing a model comprising a multiple serum biomarker seemed to be promising and critical for the detection, diagnosis, and prognosis of T2DM;^101^ however, relevant findings have not been routinely used in clinical laboratory tests. Moreover, it is challenging to characterize the broad and dynamic spectrum of serum proteins, especially in the case of low-abundance proteins. In the case of NAFLD, ApoE and lymphocyte cytosolic protein 1 (LCP1) were significantly upregulated, while IGFBP3 and vitamin D-binding protein were downregulated in patients with NASH compared with healthy subjects.^102^ In the exploration of the mechanism underlying MetS, proteomics has enabled significant advances. Evidence indicates that hyperglycemia induces metabolic changes in β cells that markedly reduce mitochondrial metabolism and adenosine triphosphate (ATP) synthesis.^103^ A study using phospho-proteomics revealed the glycogen synthase kinase 3-pancreatic and duodenal homeobox 1 axis as a key pathogenic signaling node in insulin secretion.^104^ The adipose tissue proteins identified in proteomic studies addressing diabetes and insulin resistance mainly participate in energy and metabolism, immune response/inflammation, oxidative stress, cytoskeleton, and apoptosis/cell cycle.^105–109^ Metabolic diseases including T2DM, NAFLD, and MetS comprise a series of pathway disturbances in carbohydrates, lipids, and proteins; therefore, metabolomics is quite feasible for studying these disorders 68 (Fig. 4).

Bowes *et al.*^110^ used metabolomics and genomics to better understand the relationship between near real-time population dietary assessment and wastewater-based epidemiology. Community-scale datasets were made, displaying the relationship between human behavior and dietary indicators that are measurable in municipal wastewater and allowing for the association between wastewater-borne levels of phytoestrogens and composition of gut microbiota to be established. Metabolomics, proteomics, glycomics, and microbiomics are being combined to better understand the infant gut microbiota and the metabolic impact of human milk on infants' health.^111^

### 3.4 Nutrition and microbiome

Dietary factors are the contributors to many diseases. This fact suggests that the personalization of dietary habits may have an impact on changing behavior and ultimate health outcomes.^112^ Transcript profiling has been extensively used to evaluate the possible effects of anthocyanins on obesity related gene expression in adipocytes,^113^ for biomarker identification^114^ and also for designing precise mitigation strategies especially for ready-to-eat food products.^115^ Several robust and nutrient-specific microRNAs (miRNAs) as indicators of nutritional stress have been reported in plants.^116^

Nutriproteomics is still a nascent research area, which exploits the proteomic tools to characterize molecular and cellular changes in expression of proteins and their interaction with other nutrients, as the bioavailability and functions of each nutrient including bioactive peptides and proteins can be influenced by the presence of other nutrients/compounds. Bioactive peptides and proteins derived from food in general exert multiple responses such as growth and homeostatic regulation and can even cause adverse allergic reactions in some cases.^117^ Proteomics in nutrition can identify and quantify bioactive proteins and peptides and addresses their nutritional bioefficacy.^118^ Application of proteomic techniques for determining food quality especially with respect to personalized nutrition is mainly done by analyzing the complete proteome or metabolome of food.^119^ The proteomics approach may even be used in the post-marketing surveillance of foods derived from genetically modified crops^120^ and in identification of bioactive compounds in nutraceuticals and functional foods,^121^ apart from diagnosis and vaccine/drug development.^122^ Metabolomics application to studies on dietary interventions allows a greater understanding of the effect of diet on metabolic changes, one's health and related disorders along with the relationship between the genotype and phenotype. For example, metabolomics has been used in different studies for evaluating metabolite profiles as a result of consuming fiber,^123^ tea,^124^ coffee,^125^ fish oil,^126^ and high-fat diet^127^ and a large number of metabolic perturbations have been revealed. The metabolomics approach can be used for nutritional interventions, to identify dietary biomarkers, and for the development of personalized nutrition or medicine.^128–130^

Transcriptomics, non-serum metabolomics, and genomics were tools applied by Li *et al.*^131^ to explore the mechanism and toxicological effect of inorganic arsenic exposure on the liver-microbiota-gut axis in chickens. Inorganic arsenic exposure was found to damage hepatic function-related serum biochemical indicators and alter liver transcription factors, resulting in the development of fibrosis and negatively altering the biodiversity of ileal microbiota. Liu *et al.*^132^ utilized metabolomics and microbiomics to better understand the relationship between white matter structure, gut microbiota, and metabolites in infants born with low birth weight and white matter injury. It was found that infants of this group had significant downregulation of metabolic pathways such as biosynthesis of arginine and primary bile acid, which results in white matter damage in the brain. The microbiota were found to be dysregulated, with an increase in *Klebsiella* sp. These specific bacteriota are associated with pro-inflammatory responses within the gastrointestinal tract.

Bekiares *et al.*^133^ used proteomics and microbiomics to examine the effects of sweetened dried cranberries on the urinary proteome and fecal microbiome. While there was not a statistically significant change in fecal microbiome, 22 proteins were found to have differences between pre- and post-treatment.

Also, proteomics, phosphoproteomics, and transcriptomics data demonstrated by Arumugam *et al.*^134^ revealed the mechanisms of intermittent fasting and its beneficial effects on cardiac health and disease prevention. Intermittent fasting regimens modify cyclic GMP signaling, lipid and amino acid metabolism, cell adhesion, cell death, and inflammation. It was shown that shorter intermittent fasting regimens had a larger effect on pathway alteration in comparison to longer intermittent fasting regimens.

### 3.5 Infectious diseases and sepsis

Infection is defined as a pathologic process caused by the invasion of normally sterile tissue or fluid or body cavity by pathogenic or potentially pathogenic microorganisms. Biomarkers play a role in helping to identify—or perhaps more importantly rule out—an infection. Infection is not an all-or-none phenomenon, and there are “gray areas” where one can never really be certain that an infection was present or absent. Sepsis is defined as the presence of organ dysfunction occurring as the result of a dysregulated host response to an infection. Sepsis markers such as chemokines, coagulation system markers, endotoxin, lactate, and procalcitonin are usually more helpful in ruling out than ruling in an infection. This is particularly true in critically ill patients, who often have some inflammatory response, but do not always have infection or require antibiotic administration.^135^ The application of metabolomics in infectious disease diagnostics is an evolving area of science that was boosted by the urgency of COVID-19 pandemic. Metabolomics approaches that rely on the analysis of volatile organic compounds exhaled by COVID-19 patients hold promise for applications involving a large-scale screening of population in point-of-care (POC) settings. On the other hand, successful application of mass-spectrometry to detect specific spectral signatures associated with COVID-19 in nasopharyngeal swab specimens may significantly save the cost and turnaround time of COVID-19 testing in the diagnostic microbiology and virology laboratories. Active research is also ongoing on the discovery of potential metabolomics-based prognostic markers for the disease that can be applied to serum or plasma specimens. Several metabolic pathways related to amino acid, lipid and energy metabolism were found to be affected by severe COVID-19. Tryptophan metabolism *via* the kynurenine pathway was persistently dysregulated in several independent studies, suggesting the roles of several metabolites of this pathway such as tryptophan, kynurenine and 3-hydroxykynurenine as potential prognostic markers of the disease.^136^ COVID-19 encompasses a spectrum of varying phenotypes where customized therapy may help more and harm less. Rello *et al.* (2020) have described 5 phenotypes ranging from the most benign (phenotype 1) to increasing respiratory distress and hypoxemia (phenotypes 2 and 3) and acute respiratory distress syndrome (ARDS) (phenotypes 4 and 5).^137^ IL-6 has been suggested as a differentiating feature between phenotypes 2 and 3, and procalcitonin as a characteristic feature of phenotype 5. Defining phenotypes based on underlying risk factors, clinical and radiological features and biomarkers may help predicting the need for ICU admission and optimizing therapy. One study assessed the changes in biomarkers with supportive therapy and a variable combination of abidol, lopinavir/ritonavir and methylprednisolone.^138^ After treatment, IL-2R, IL-6, TNF-α, and CRP levels decreased significantly, followed by IL-8, IL-10, and PCT. CD4+ and CD8+ T lymphocytes increased significantly but B lymphocytes and natural killer cells showed no changes. Serum ferritin also did not decrease significantly. D-dimer levels have been recommended as a part of the risk stratification criteria to decide anticoagulation.^139^ Treatment with low molecular weight heparin (LMWH) is associated with reduction in levels of d-dimer and fibrin degradation products and also in IL-6 levels suggesting a potential anti-inflammatory effect.^140^

The lipidomics data revealed how the lipidome controls the immune response and its effect on sepsis severity depending on the COVID-19 status. Expression of inflammatory hubs responsible for restricting inflammation, such as ChoE-18 : 3, LPC-O-16 : 0, and PC-O-30 : 0, is decreased in patients with sepsis from COVID-19, while expression of inflammatory hubs responsible for enhancing inflammation, such as sPLA2, PGD2, and 12-HETE, is increased in patients with sepsis from COVID-19 as reported by Meng *et al.*^141^ Data from a multi-omics approach using proteomics, metabolomics, and lipidomics were applied to build a workflow to predict aggravation of COVID-19 symptoms of patients in the ICU. Two proteins (CCL7 and CA14), as well as one lipid (HexCer 18 : 2; O2/20 : 0), were identified as short-term predictors of worsening COVID-19 progression in ICU patients as described by Kugler *et al.*^142^

Also, metabolomics and microbiomics profiling has been utilized recently by Bosnjak *et al.*^143^ to characterize the fecal environment of patients with hospital acquired diarrhea before and after receiving antibiotic treatment in relation to the *C. difficile* infection status. It was found that *C. difficile* infection alters metabolic markers that result in antibiotic-associated dysbiosis and proliferation of opportunistic bacteria. Transcriptomics and proteomics were utilized by Noszka *et al.*^144^ to better understand the HP1021 regulon and its relationship with *H. pylori.* HP1021 controls the response of *H. pylori* to oxidative stress, as well as DNA uptake and carbohydrate metabolism, directly.

Zeng *et al.*^145^ reported that post analysis integration of transcriptomics and proteomics data of synovial cells and fibroblasts has been performed to better understand inflammation pathways in which the drug celastrol impacts rheumatic arthritis cells by modulating inflammation, inhibiting chemokine pathways and osteoclast differentiation, and promoting synovial cell apoptosis.

### 3.6 Neurodegenerative diseases and inflammation

Biomarkers for neurodegenerative diseases are needed to improve the diagnostic workup in the clinic but also to facilitate the development and monitoring of effective disease-modifying therapies. Positron emission tomography methods for detecting amyloid-β and tau pathology in Alzheimer's disease have been increasingly used to improve the design of clinical trials and observational studies. In recent years, easily accessible and cost-effective blood-based biomarkers used for detecting the same Alzheimer's disease pathologies have been developed, which might revolutionize the diagnostic workup of Alzheimer's disease globally. Relevant biomarkers for α-synuclein pathology in Parkinson's disease are also emerging, as well as blood-based markers of general neurodegeneration and glial activation.^146^ Several fluid biomarkers of neurodegeneration have recently emerged. Blood-based assays reveal brain α-synuclein pathology and would considerably facilitate studies in larger populations, but skin biopsies might also provide an effective alternative. Analysis of CSF is likely to be central in this process because the levels of brain-derived proteins are much higher in CSF than in blood, where brain-derived molecules are diluted in a complex matrix of abundant plasma proteins, such as albumin and immunoglobulins. Currently, the most promising marker of neurodegenerative disease is the neurofilament light (NfL), which can be measured in both CSF and blood. This biomarker reflects axonal degeneration and injury, irrespective of cause, and the levels are especially increased in amyotrophic lateral sclerosis, frontotemporal dementia, and atypical parkinsonian disorders (that is, progressive supranuclear palsy, multiple system atrophy (MSA) and corticobasal degeneration).^147,148^ However, NfL levels are also increased in Alzheimer's disease, and studies on autosomal dominant Alzheimer's disease show that the rate of change in blood NfL increased already about 15 years before symptom onset.^149^ Importantly, higher levels of NfL are associated with faster disease progression and higher brain atrophy rates in most neurodegenerative disorders.^147,149^ As a result, NfL can be regarded as a measure of the intensity of ongoing neurodegeneration. In several brain diseases, including multiple sclerosis and spinal muscular atrophy, effective disease-modifying treatments can normalize NfL levels, and reduction in NfL levels is associated with the clinical effectiveness of the treatment.^150,151^

Regarding inflammatory diseases, proteomics of synovial fluid and plasma revealed that there is a mild time series pattern of expression during osteoarthritis progression as reported by Anderson *et al.*^152^ At the initial induction of osteoarthritis, there was a decrease in proteins responsible for signal transduction and regulation of signaling events at day 10, followed by an increase at day 63. On day 10, there was also an initial response in proteins that are responsible for conducting immune system responses. Overall, proteomics data showed that an EV protein cargo is more important than a small non-coding RNA cargo during osteoarthritis progression.

Transcriptomics, proteomics, and phosphoproteomics methods were applied by Wei *et al.*^153^ to better understand the inflammatory and metabolic pathways involved in diabetic kidney disease progression. Pathways found to have a signficant effect on the disease include lipid metabolism, fatty acid metabolism, glycolysis, cell cycle regulation, phagocytosis and apoptosis regulation, and inflammatory response regulation. Genes such as ALOX15, known to enhance hypertrophy, fibrosis, and pro-inflammatory gene formation, and SERPINA1E, known to inhibit liver gluconeogenesis, were found to be downregulated, negatively affecting the identified pathways. Integrated transcriptomics and proteomics data were utilized by Pascual-Alonso *et al.*^154^ to analyze human fibroblasts to study the molecular consequences of mutated MECP2 in individuals with Rett Syndrome. It was revealed that due to a loss in function of the mutated MECP2 protein, other genes and proteins responsible for neuronal development are downregulated, resulting in neuronal dysregulations, such as cytoskeletal organization, vesicular activity, and mRNA processing.

### 3.7 Respiratory diseases and cancer

Asthma is one of the most prevalent chronic airway diseases characterized by airway hyper-responsiveness, inflammation, and mucus secretion. It is one of the most prevalent chronic airway diseases, affecting approximately 339 million individuals worldwide, globally killing more than 1000 daily, and its incidence rises each year.^155^ Among the largest investigations is a European genome-wide association (GWAS) study on allergic disease susceptibility conducted in 360 838 subjects, which identified 136 genetic variants to be associated with allergic disorders, including asthma, implicating 132 nearby genes from 99 loci.^156^ Large-scale epigenome-wide association (EWAS) studies indicated that environmental exposures such as prenatal smoking and air pollution were associated with changes in DNA methylation patterns of several asthma-related genes.^157,158^ The transcriptome profiles of different tissues/cells have provided significant insights into the role of gene expression in the asthma disease process. A study in healthy controls and mild, moderate, and severe asthmatics showed that CD3+ T cells isolated from sputum and bronchoalveolar lavage fluid (BALF) had a distinct transcriptome profile from endobronchial epithelial brushings.^159^ Airway proteome profiles in asthma patients can be associated with different phenotypes. The importance of proteomics is that it can represent the active cellular state of different tissues/cells. However, adequate attention should be directed to tissue-associated proteome differences in comparative and possibly longitudinal studies. Sputum proteomics showed that 10 out of 1129 proteins were significantly different between four previously established clinical asthma clusters.^160^ Moreover, the sputum proteome profiles of adult asthmatics were distinguished between current, ex-smokers, and nonsmokers.^161^ Most conducted metabolomics studies in asthmatics focused on investigating metabolomics profiles in comparison to healthy controls and other respiratory disorders such as chronic obstructive pulmonary disease (COPD) or distinguishing different asthma phenotypes. The main identified metabolites across different studies from various body compartments were related to immune reactions, inflammatory processes, tricarboxylic acid cycle, oxidative stress, hypoxia, and lipid metabolism pathways.^162^ One of the emerging metabolomics techniques in asthma research is the measurement of volatile organic compounds (VOCs) in exhaled breath (breathomics).^163,164^

Regarding multi-omics analysis Li *et al.*^165^ demonstrated that serum proteomics and metabolomics revealed the underlying pathogenesis of severe community-acquired pneumonia, which involves enhancing inflammatory pathways including Hippo and PI3K/Akt, leading to tissue damage resulting from overactivation of inflammatory signals, while decreasing immunoglobulins and suppressing the overall function of humoral immunity. Metabolomics revealed suppression of lipid metabolism with enhanced glycolysis and lactate production, resulting in disordered lipid metabolism. Qu *et al.*^166^ integrated transcriptomics and genomics data of lung tissues to better understand the impact and underlying mechanisms of CD93 on prognosis of lung squamous cell carcinoma. Increased expression of CD93 has shown to result in upregulation of cell adhesion and angiogenesis pathways, suggesting that CD93 plays a significant role in the formation of the capillary network of primary tumors and increasing the likelihood of lymph node and distant metastasis. Increased expression of CD93 in endothelial cells also resulted in T cell dysfunction, inducing the local immune tolerance. Also Wang *et al.*^167^ integrated transcriptomics to map the cellular subpopulations within the immune microenvironment of the brain to build a predictive model for intracranial aneurysms. M1/M2 type macrophages were found to play a critical role in intracranial aneurysm development, along with the presence of RGS1, which activates and progresses inflammatory signaling. Xie *et al.*^168^ used transcriptomics and genomics methods to develop a greater understanding of the effects of G protein-coupled receptors on lung adenocarcinoma and create a prognostic model that tests responses of patients to immunotherapy and sensitivity to first-line drugs.

Table 1 presents a summary of representative multi-omics studies described above.

**Table 1:** Primary studies regarding multi-omics analysis

Focus	Type of omics	Biofluid	Analytical approach	Biomarkers	Species sample size	Ref.
Baseline health monitoring	Peripheral blood, plasma, and serum	Transcriptomics, proteomics, and metabolomics	RNAseq	Primary circadian pacemaker (PER1), inflammatory molecules (C2, C9, IL5), pro-inflammatory cytokines (IP10, IL1, IL1R1)	Human, *n* = 105	77
Baseline health monitoring	Blood, saliva, stool, and urine	Metabolomics, proteomics, and microbiomics	RNAseq, SRM, and use of public databases	Gamma-glutamyltyrosine	Human, *n* = 108	78
Baseline health monitoring	Heart tissues for intermittent fasting	Proteomics, phosphoproteomics, and transcriptomics	Use of public databases, LC-MS, immunoblot assay	Metabolic enzymes (PFKFKB1/2, HK1, PCK2, AKT1, mTOR, MAPK9), metabolic proteins (AHSG, SRSF1, APOE, ACAD8, PKM), phosphoproteins (CDK, PDK, AMPK, PKA, EGFR), and transcription factors	Mouse	134
Environmental exposure	Blood serum and fecal matter	Transcriptomics, serum non-targeted metabolomics, and genomics	ELISA, colorimetry, western blot assay, RNAseq, and LC-MS	Arsenic-induced liver fibrosis genes (TGFB1, ACTA2, COL1A1), vital oxidative stress response genes (SOD1, GPX4, GST gene family), steatosis-related genes (PPARG, FABP5, ACOX1) liver apoptosis markers (BCL-2, COG gene family), inflammation response genes (NF-kB, IL-β)	Chickens, *n* = 40	131
Environmental exposure	Urine and left lung tissue for cigarette smoke exposure	Transcriptomics, proteomics, lipidomics, metabolomics	HRMS/MS, UHPLC-MS/MS, and use of public databases	mRNA (C1qtnf4), miRNA (mmu-mir-146a, mmu-miR02137, mmu-miR-21a), polyamines (putrescine, *N*-acetyl-putrescine, acetyl-spermidine), proteins (Sod2, cat, Txnrd1, Atox1, G6pdx), and enzymes	Mouse	90
Environmental exposure	Blood, liver and brain tissues for benzalkonium chloride exposure	Lipidomics and transcriptomics	HILIC-IM-MS, UHPLC-MS/MS, RNAseq, and use of public databases	Sterol precursors (7- and 8-DHC, 7-DHD, desmosterol, lanosterol), lipids (DGs, TGs, HexCers, and Cers), proteins (SREPBs), and sterol biosynthesis genes (Hmgcs2, Idi1, Cyp51)	Mouse	91
Metabolic and inflammatory diseases	Rheumatoid arthritis fibroblasts and synovial fibroblasts	Proteomics, transcriptomics and single-cell transcriptomics	Use of public databases and network construction analysis	Multi-pathway (P13K/AKT, Th17, MAPK, TNF, JAK-STAT)	Cultured cells	145
Metabolic and inflammatory diseases	Synovial fluid and plasma for osteoarthritis	Proteomics	Reporter gene assay	Synovial fluid-derived EVs (stabilin 1, perilipin 4, apolipoprotein C-IV)	Horse, *n* = 4	152
Metabolic and inflammatory diseases	Urine, blood, and kidney tissues for diabetic kidney disease	Transcriptomics, proteomics, and phosphoproteomics	LC-MS/MS, RNAseq, and HPLC	Genes (ALOX15, COL19A1, CXCL3, GKN3, UGT1A2), proteins (ALOX15, SERPINA1E, AKR1C18, SLCO1A1, UGT1A1), phosphorylated proteins (ENO1, MIOX, NQO1, LRRFIP1)	Mouse	153
Nutrition	Water samples for wastewater-based epidemiology	Metabolomics and genomics	LC-MS/MS	Phytoestrogen metabolites (genistein, daidzein, enterolactone), bacterial taxa (*Bifidobacterium, Blautia, Romboutsia, Clostridium, Dorea, Subdoligranulum, Intestinibacter, Eubacterium, Bacteroides, Prevotella, Senegalimassilia, Roseburia, Slackia*)	Phytoestrogen	110
Nutrition	Blood, breast milk, fecal matter, urine, and saliva	Metabolomics, proteomics, and peptidomics	NMR, Qubit dsDNA high-sensitivity assay, and LC-MS/MS	Analysis not completed	Human, *n* = 200 mother–infant dyads	111
Nutrition	Urine and fecal matter	Proteomics and microbiomics	LC-MS, use of public databases, and DMAC assay	Urinary proteins (VIP36, PIK3IP1, uromodulin), microbiota (*Bacteroidetes, Firmicutes, Akkermansia*)	Human, *n* = 10	133
Infectious diseases and sepsis	Fecal matter for Hospital-acquired diarrhea	Metabolomics and microbiomics	RNAseq, GC-MS, SCFA analysis, and use of public databases	Proline Stickland fermentation by-products (5-aminovaleric acid, isovalerate, isobutyrate), l-leucine and l-valine Stickland fermentation by-products (4-MPA/l-leucine ratio)	Human, *n* = 169	143
Infectious diseases and sepsis	Blood and urine for COVID-19	Lipidomics and proteomics	LC-MS, DESI-MS, ELISA assay, and O-link proximity extension assay	Phospholipases (sPLA2-ILA, PLA2G2D, PGE2, 5(6)-DHET), eicosanoids (5- and 15-HETE, DHETs, DiHOMEs), and phospholipids (ChoE-18 : 3, LPC-O-16 : 0, and PC-O-30 : 0)	Human	141
Infectious diseases and sepsis	Not described	Transcriptomics and proteomics	LC-MS/MS, ChIP-seq analysis, and EMSA	HP1021	Cultured *Helicobacter pylori*	144
Infectious diseases and sepsis	Blood for SARS-CoV-2	Proteomics, metabolomics, and lipidomics	LC-HRMS, LC-MS/MS, and FIA-MS/MS	Lipid metabolites (thyroxin, tryptophan, and kynurenine), lipids (LPC, LPE, PC, and hexosylceramide classes), and proteins (CCL7, CA14, and HexCer)	Human patients, *n* = 32	142
Neurodegenerative diseases and inflammation	Intracranial aneurysm fibroblasts and macrophages	Transcriptomics	Use of public databases, network construction analysis, western blot assay, and RNAseq	Ccl5, Ptgds, Spp1, Fos, Cfb, Rgs6, Slamf8, Nbl1, Ndrg2, Tmem158, RGS1. CD68, CXCL1, MCP-1, TNF-α	Cultured cells, *n* = 97	167
Neurodegenerative diseases and inflammation	Fecal matter for white matter disease	Metabolomics and microbiomics	Use of public databases, LC-MS/MS, and MRI	Microbiota	Human	132
Neurodegenerative diseases and inflammation	Rett syndrome fibroblasts	Transcriptomics and proteomics	RNAseq, TMT-MS, and public databases	Transcription factors (CREB1, SRF, HEYL, GLIS2, NFATC4, JUN), DE genes (MYO1C, HARS2, MECP2), and DEP genes (APPL2, CNPY4, CTSC, REPS1, CNN1)	Human, *n* = 49	154
Respiratory diseases and cancer	Blood serum for community-acquired pneumonia	Proteomics and metabolomics	Use of public databases, ELISA, and LC-MS/MS	Hippo signaling pathway (ASS1, SAA2 SFTPB, CRP, LBP, CETP, ITIH1, and LDHA), metabolic pathways (TKT, GPI, LCAT, APOA1, APOA2, APOA4, APOC1, APOL1), and inflammation-related pathways (IGHG1, IGHG2, IGHM, TLR, and PI3K/Akt)	Human, *n* = 161	165
Respiratory diseases and cancer	Lung tissue for lung squamous cell carcinoma	Transcriptomics and genomics	Use of public databases and network construction analysis	CD93	Human, *n* = 61	166
Respiratory diseases and cancer	Lung adenocarcinoma	Transcriptomics (single-cell and bulk) and genomics	Use of public databases and network construction analysis	G protein-coupled receptor-related genes (CCL20, DDIT4, GPX3, BEX5, AKAP12, DSG2, SERPINH1, LDHA, DNAJB4, and DOCK4)	Cultured cells	168

## 4 Diagnostic value of biofluids

### 4.1 Overview

**Fig. 5:**
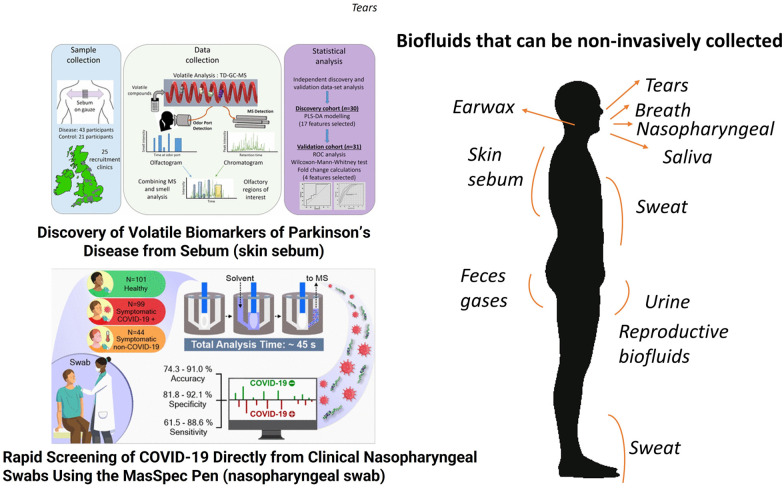
The composition and diagnostic value of blood and some biofluids that can be collected non-invasively are indicated in the right side of the figure; saliva, sweat, urine, nasopharyngeal mucus, breath, and skin sebum and urine are explored in this review. On the left side of the figure, illustrative examples show that skin sebum obtained from t-shirts was successfully used to diagnose Parkinson's disease,^183^ and nasopharyngeal mucus sampled directly from the swab and analyzed using a mass spectrometer was used to screen for COVID-19 infection.^185^ Adapted with copyright permission from ACS Publications.

Blood is a commonly used biofluid for omics analyses. It is composed of two parts: a cellular component consisting of red and white blood cells and platelets, and a liquid carrier, called plasma. Plasma accounts for approximately 50–55% of blood volume, with blood cells (erythrocytes, leukocytes and platelets) accounting for the remaining portion. Plasma is obtained from a blood sample, if anti-coagulants are introduced, by simply centrifuging the sample and removing or decanting the most buoyant (non-cellular) portion. If no anticoagulant is added and the blood is allowed to clot, the supernatant fluid is called the serum, which is less viscous than plasma and lacks fibrinogen, prothrombin, and other clotting proteins. Both plasma and serum are aqueous solutions (about 95% water) containing a variety of substances including proteins and peptides (such as albumins, globulins, lipoproteins, enzymes, and hormones), nutrients (such as carbohydrates, lipids and amino acids), electrolytes, organic wastes and a variety of other small organic molecules suspended or dissolved in them. Based on current analytical techniques the primary difference between serum and plasma appears to lie in the compounds involved in the clotting process, although modest discrepancies in the relative distribution of some compounds between these pools have also been reported. Serum is a primary carrier of small molecules in the body and its chemical composition has been studied in the last 70 years.^169–171^ The successful identification of biomarkers in blood, serum or plasma has a long history, which started with blood typing to guide blood transfusions,^172^ followed by newborn metabolic screening for the early detection of metabolic diseases,^173^ analysis of serum prostate-specific antigen for the early detection of prostate cancer^174^ and dozens of other applications. Dozens of proteins have received FDA approval for clinical practice and most of them are for cancer diagnosis.^175^ Human metabolites, mostly present in blood, plasma, or serum, serve as valid clinical markers and are currently compiled in the Mayo Clinic test catalog (https://www.mayocliniclabs.com/test-catalog/), LabCorp test menu (https://www.labcorp.com/test-menu/search), and Quest Diagnostics Test Directory (https://testdirectory.questdiagnostics.com/test/home). The word metabolomics in the U.S. National Library of Medicine at Clinical Trials retrieved 962 studies (https://clinicaltrials.gov/) and an almost equal number of completed or recruiting clinical studies. Saliva is a complex bodily fluid consisting of *ca.* 99% water, inorganic and organic substances and a variety of proteins such as enzymes, mucus and glycoproteins.^176^ The potential of salivary inorganic constituents, antioxidants, hormones, antibodies and antigens as biomarkers in the diagnosis of several oral and systemic diseases is expanding and has been recently reviewed.^177^ In oral diseases, saliva has been used to detect oral cavity cancer, dental caries, periodontal disease, and oral dryness. In systemic diseases (*i.e.*, diseases that affect the entire body), saliva has been demonstrated to have strong correlations with plasma or serum for many cell components. Examples include the monitoring of hormone levels, pregnancy, risks for preterm labor, psychological disorders, neurological disorders, immune system status, smoking status, virus infections, malaria and nutritional status.^177–179^ Sweat is a slightly acidic biofluid composed mainly of water (99%), electrolytes (*e.g.*, sodium, chloride, and potassium), urea, pyruvate, and lactate. Proteins, peptides, amines, amino acids, and metal ions in smaller concentrations are also found in this biofluid in addition to inhibitors, antigens, antibodies, and a variety of xenobiotics such as drugs, cosmetics, and ethanol. These substances are stored in the sweat glands and secreted into the sweat. At the epidermis surface, partial selective reabsorption of sodium and chloride takes place during transportation, which results in hypotonicity of the secreted sweat in healthy individuals.^180^ Diseases can change sweat composition either by altering the concentration of common components or by forming new components that act as biomarkers for diseases.^181^ Except for the case of some high molecular weight proteins, which reach sweat by different intracellular storage mechanisms in particular situations, most sweat components are small molecules resulting from metabolic pathways; therefore, biomarkers characterized so far are mostly metabolites. Besides sweat, the human skin has sebaceous glands responsible for the continuous production of sebum. Human sebum consists of squalene, esters of glycerol, wax, and cholesterol, as well as free cholesterol and fatty acids. Triacylglycerides and fatty acids, taken together, account for the predominant proportion (57.5%), followed by wax esters (26%) and squalene (12%). The metabolic pathways regulating its composition and secretion rate are far from complete understanding.^182^ Recently, skin sebum has shown promising results as a sample for the diagnosis of neurodegenerative diseases (Fig. 5).^183,184^

Urine is the most widely used biological specimen, apart from blood. Interest in using urine for diagnostic purposes arises from the fact that it is a rich source of disease biomarkers, and the sample can be obtained noninvasively. Unlike blood, urine has a relatively low concentration of proteins and many low molecular weight compounds (metabolites); hence metabolomics studies of urine are relatively simple in terms of both sample preparation and analysis. Metabolomics studies with nuclear magnetic resonance and mass spectrometry can measure the concentration of more than 3 000 chemical compounds in the urine, providing possible chemical signatures of different diseases and health.^186^ Urinary proteomics and metabolomics studies show altered signatures in patients with gastrointestinal disorders and cancer compared to healthy controls. All of these disorders may include the alteration of urinary metabolites in association with the gastrointestinal microbiota and possibly dysbiosis, especially in chronic conditions.^187^ Nasal secretions originate mostly from submucosal glands and goblet cells. Mucus is composed of water (95%); glycoproteins (2%); albumin, immunoglobulins, lysozyme, lactoferrin and other proteins (1%); inorganic salts (1%); and lipids (<1%).^188^ In recent years, investigations on upper airway mucosa inflammation in response to inoculation with bacterial or viral pathogens,^189,190^ allergen challenge,^191–193^ or exposure to environmental pollutants^194–196^ have focused on the detection of minute amounts of cytokines and inflammatory mediators. Recently, COVID-19 diagnosis in nasal secretion based on small molecules has been reported by more than one mass spectrometry method,^185,197^ as described in Fig. 5.

Exhaled breath and breath condensate contain inorganic and organic compounds, as well as aerosols in the form of water vapor and particles. Focusing on the gas phase, breath contains diverse inorganic species and several hundred volatile organic compounds (VOCs) of diverse chemical nature, the latter being present only in trace quantities.^198^ Breath analysis has received unprecedented attention recently in relation to the severe acute respiratory syndrome associated with the COVID-19 pandemic.^199^ The potential for exhaled breath to either detect this airborne virus directly or to diagnose infection is currently being investigated as a comfortable alternative to existing approaches that collect mucus secretions. While no breath test has yet been translated to patients for the reliable detection of the infection, studies have reported potential breath-borne VOC biomarkers detected *via* gas chromatography ion mobility spectrometry (GC-IMS) or specific breathprints using proton transfer reaction time-of-flight mass spectrometry (PTR-TOF-MS).^200^ Several breath tests exploit exogenous compounds, such as the well-known and widely implemented breath alcohol ‘breathalyser’ test, as used in law enforcement to identify drink-drivers,^201^ breath tests for hypolactasia,^202^ and *Helicobacter pylori* infection.^203^ In the latest Breath Biopsy Conference, advancements in the collection and analysis of volatile organic compounds in exhaled breath for the diagnosis of asthma, cirrhosis, cancer, and tuberculosis have been documented.^204^

### 4.2 Limitations of using biofluids for molecular phenotyping and declining health

In blood and related biofluids (serum and plasma) the concentration of the average metabolite normally can vary by about ±50%, with some metabolites varying by as much as ±100% (such as l-lactic acid, l-glutamine, glycine). These relatively large ranges of metabolite concentrations are challenging to baseline measurements and are due to several factors, including age, gender, genetic background, diurnal variation, health status, activity level, and diet.^169,205^ Saliva is a viscous fluid due to the presence of mucopolysaccharides and mucoproteins. The difficulty in measuring precise volumes of viscous saliva samples can decrease the accuracy of analytical measurements. Due to the low concentrations of compounds in saliva, assays with high sensitivity and low limit of detection are required. The components of saliva may depend on the area in the oral cavity in which the sample is collected as well as the collection methods (unstimulated *vs.* stimulated). Also, oral diseases such as gingivitis, change in pH and other potential interferences such as smoking, fasting and hydration level can also influence the levels of the biomarkers of interest.^206^ There are relatively large variations in metabolite concentrations between different individuals because many salivary components play multifunctional roles and, in some cases, have overlapping functions. For example, glutathione, ascorbic acid, and uric acid are all antioxidants. Amylases, cystatins, histatins, mucins and peroxidases are all involved in antibacterial activity in the oral cavity. Diurnal fluctuation, seasons (temperature) and other intra-individual inconsistency may also have to be considered.^175^ Regarding sweat tests, the main limitations are related to the high cost and special care required for the collection patches and the lack of automation.^207^ There is also the need to normalize sampled volume by reference compounds such as sodium.^208^

Urine has long been a “favored” biofluid among metabolomics researchers. It is easy to obtain in large volumes and chemically complex. However, this chemical complexity has also made urine a particularly difficult substrate to fully understand. As a biological waste material, urine typically contains metabolic breakdown products resulting from a wide range of foods, drinks, drugs, environmental contaminants, endogenous waste metabolites and bacterial by-products. Many of these compounds are poorly characterized and understood. Besides this, diagnostic compounds in urine present diverse confounding effects, such as diet variations, large inter- and intra-individual variations, variations induced by sample collection, handling and storage and inconsistency in data extraction, interpretation, and analytical methods. Also, effects of the kidney function and the metabolic function of the body, which may affect secretion and reabsorption of the circulating metabolites may confound the final results.^209,210^ Biological markers in nasal secretions provide valuable information on nasal pathophysiology and as a health indicator, this biofluid has so far mainly been explored for infectious disease diagnostics. Published data on biomarker concentrations in nasal fluids are inconsistent mainly due to different sampling techniques.^211^ Regarding breath analysis, a major challenge is the sensitive detection of individual compounds. There are also technological limitations associated with reliably capturing breath and the analytical intricacy of extracting potential biomarkers from complex datasets.^198,212^ The lack of standardization in breath analysis has led to a limited alignment of results between independent studies employing different approaches.^213^ The successful development and implementation of the nitric oxide breath test for asthma represents an example of diagnosis using breath analysis.^214^

## 5 Examples of databases focused on human disease and exposure

Currently, 500 000 chemicals (synthetic and endogenous small molecules) can be found in our bodies, in our food or in the environment. This information is catalogued into publicly accessible databases, and those listed below include molecular features related to diseases. Libraries can be commercial but there are extensive open-access databases, such as *m/z* Cloud (https://www.mzcloud.org/) and the Human Metabolome Database (described below in more detail), containing hundreds of thousands of mass spectra that provide chemical structural information of endogenous and exogenous (synthetic) compounds.

Marker DB: this database is freely available and attempts to consolidate information on all known clinical and a selected set of pre-clinical biomarkers into a single resource. MarkerDB includes five major types of biomarkers (condition-specific, protein, chemical, karyotypic and genetic) and four biomarker categories (diagnostic, predictive, prognostic and exposure). Information compiled include biomarker names and synonyms, associated conditions or pathologies, detailed disease descriptions, detailed biomarker descriptions, biomarker specificity, sensitivity and ROC curves, standard reference values (for protein and chemical markers), variants for SNP (single nucleotide polymorphisms) or genetic markers, sequence information (for genetic and protein markers), molecular structures (for protein and chemical markers), tissue or biofluid sources (for protein and chemical markers), chromosomal location and structure (for genetic and karyotype markers), clinical approval status and relevant literature references. Users can browse the data by conditions, condition categories, biomarker types, biomarker categories or search by sequence similarity through the advanced search function. Currently, the database contains 142 protein biomarkers, 1089 chemical biomarkers, 154 karyotype biomarkers, and 26 374 genetic markers. These are categorized into 25 560 diagnostic biomarkers, 102 prognostic biomarkers, 265 exposure biomarkers and 6746 predictive biomarkers. Collectively, these markers can be used to detect, monitor, or predict 670 specific human conditions, which are grouped into 27 broad condition categories (https://markerdb.ca).

Online Mendelian Inheritance in Man (OMIM) is a comprehensive compendium of human genes and genetic phenotypes. The full text and referenced overviews in OMIM contain information on all known Mendelian disorders and over 15 000 genes. OMIM focuses on the relationship between phenotype and genotype. It is updated daily, and the entries contain many links to other genetics resources. OMIM contains nearly 4800 single gene disorders and traits as well as 5800 phenotypes for which the molecular basis is known. https://www.omim.org/.

OMMBID or the On-Line Metabolic and Molecular Basis to Inherited Disease is a subscription-based online book/encyclopedia describing the genetics, metabolism, diagnosis, and treatment of hundreds of metabolic disorders contributed by hundreds of experts. It also contains extensive reviews, detailed pathways, chemical structures, physiological data, and tables that are particularly useful for clinical biochemists (https://ommbid.mhmedical.com/book.aspx?bookID=2709#225069394).

METAGENE is a knowledge base for genetic metabolic disorders providing information about the disease, genetic cause, treatment and the characteristic metabolite concentrations or clinical tests that may be used to diagnose or monitor the condition. It has data on 1150 genetic diseases (https://www.metagene.de/).

The PharmGKB database is a central repository for genetic, genomic, molecular, and cellular phenotype data and clinical information about people who have participated in pharmacogenomics research studies. The data include, but not limited to, clinical and basic pharmacokinetic and pharmacogenomic research in the cardiovascular, pulmonary, cancer, pathway, metabolic and transporter domains. Its aim is to aid researchers in understanding how genetic variation among individuals contributes to differences in reactions to drugs. PharmGKB contains searchable data on genes (>20 000), diseases (>3000), drugs (>2500) and pathways (235). It also has detailed information on 470 genetic variants (SNP data) affecting drug metabolism (https://www.pharmgkb.org/).

SMPDB (The Small Molecule Pathway Database) is an interactive, visual database containing more than 30 000 small molecule pathways found in humans only. The majority of these pathways are not found in any other pathway database. The pathways include metabolic, drug, and disease pathways (https://smpdb.ca/).

Serum metabolome database: the serum metabolome database is integrated into the Human Metabolome Database (HMDB), allowing users to browse the data in different views, metabolites, concentrations, and diseases. It is a freely available electronic database containing detailed information about 4651 small molecule metabolites found in human serum along with 10 895 concentration values. The data tables may be sorted and searched by concentration values and ranges. The information includes literature and experimentally derived chemical data, clinical data, and molecular/biochemistry data. Each MetaboCard entry contains more than 110 data fields and many of them are hyperlinked to other databases (KEGG, PubChem, MetaCyc, ChEBI, PDB, Swiss-Prot, and GenBank) (https://www.serummetabolome.ca).

Exposome-explorer is the first database dedicated to biomarkers of exposure to environmental risk factors for diseases. It contains detailed information on the nature of biomarkers, the population studied, biospecimen(s) used, biomarker concentrations and the reference publication(s) (https://exposome-explorer.iarc.fr/).

The METLIN metabolite database is a repository with full access by subscription for mass spectral metabolite data. METLIN is searchable by compound name, mass, formula, or structure. It contains over 930 000 molecular standards as of December 2023. METLIN contains MS/MS, LC/MS and FTMS data that can be searched by peak lists, mass range, biological source, or disease (https://metlin.scripps.edu/).

FooDB is the world's largest and most comprehensive resource on food constituents, chemistry, and biology. Each chemical entry in the FooDB contains more than 100 separate data fields covering detailed compositional, biochemical, and physiological information (obtained from the literature). Users can browse or search FooDB by food source, name, descriptors, function, or concentrations (https://foodb.ca/).

DrugBank: the latest release of the database (version 5.0) contains 9591 drug entries, including 2037 FDA-approved small molecule drugs, 241 FDA-approved biotech (protein/peptide) drugs, 96 nutraceuticals and over 6000 experimental drugs. Additionally, 4270 non-redundant protein (*i.e.*, drug target/enzyme/transporter/carrier) sequences are linked to these drug entries. Each DrugCard entry contains more than 200 data fields with half of the information being devoted to drug/chemical data and the other half devoted to drug target or protein data. The DrugBank Online website is available to the public as a free-to-access resource. However, use and re-distribution of content from DrugBank Online or the underlying DrugBank Data, in whole or part, and for any purpose requires a license. Academic users can apply for a free license for certain use cases while all other users require a paid license (https://www.drugbank.com/).

T3DB: the Toxin and Toxin Target Database (T3DB) or soon to be referred as the Toxic Exposome Database is a unique bioinformatics resource that combines detailed toxin data with comprehensive toxin target information. The database currently houses 3678 toxins described by 41 602 synonyms, including pollutants, pesticides, drugs, and food toxins, which are linked to 2073 corresponding toxin target records. Altogether there are 42 374 toxin and toxin target associations. Each toxin record (ToxCard) contains over 90 data fields and holds information such as chemical properties and descriptors, toxicity values, molecular and cellular interactions, and medical information. This information has been extracted from over 18 143 sources, which include other databases, government documents, books, and scientific literature. The focus of the T3DB is on providing mechanisms of toxicity and target proteins for each toxin (https://www.t3db.ca/).

ContaminantDB: the contaminantDB is a unique bioinformatics resource that combines detailed contaminant data from different online references and databases on contaminants. The database currently houses 54 249 compounds. It is both modelled after and closely linked to the Human Metabolome Database (HMDB) and DrugBank. The databases and sources used to gather contaminant data include IARC carcinogens group 1, 2A, 2B, 3 and 4, DrugBank drugs and metabolites, disinfection by-products, MyExposome Chemicals, ToxCast and Tox21 chemicals, EPA high production volume chemicals, OSHA hazardous chemicals, Clean Air Act Chemicals, T3DB toxins, ECHA substances of high concern, DEA chemicals, EPA endocrine screening chemicals, EAFUS chemicals, and OECD high production volume chemicals (https://contaminantdb.ca/).

BioTransformer: BioTransformer 3.0 is a software tool that predicts small molecule metabolism in mammals, their gut microbiota, and the soil/aquatic microbiota. Moreover, it can also assist in the identification of metabolites, which is based on the metabolism prediction (https://biotransformer.ca/).

### 5.1 Metabolome annotation in databases

Metabolomics too often relies on library matches to data from previously identified standards for compound identification and leaves many, potentially important, compounds in datasets unidentified. The “dark matter” metabolome can be defined as the metabolites not yet curated in available databases. These can be either not extracted and/or not identified using standard analytical methods or are lost/transformed during extraction. Even though the latest version of the Human Metabolome Database – HMDB (version 5.0) lists 220 945 metabolite entries (almost two-fold increase from version 4.0), the actual number of human metabolites is expected to be about 10 times higher. Note that the HMDB does not include anthropogenic compounds (*e. g.* pollutants), but it harbors large quantities of predicted MS/MS and GC-MS reference spectral data and predicted (physiologically feasible) metabolite structures.^215^

Developing new analytical methods and technology could provide new insights into the dark metabolome, reduce information loss, and generate fresh insights and new knowledge in many fields, including animal health, biomedicine, disease diagnostics, environmental and food science, physiology, pharmacology, toxicology and zoology. In short, illuminating the dark metabolome could be a leap forward in the molecular characterization of biological systems.^30,215^

One of the approaches to tackle the dark metabolome is the use of *in silico* predictive tools and mining existing data for building evidence for the presence of small molecules in complex samples using libraries of calculated chemical properties and associated matching to experimental data using multiple molecular attributes (*i.e.*, multiattribute matching). Therefore, libraries of chemical properties derived computationally can replace libraries derived from authentic standards for compound identification under specific conditions, for example, when the evidence of presence is strong enough and confidence is high enough to support the intended application.^216^

## 6 Mass spectrometry instrumentation for omics analyses

### 6.1 Benchtop MS instrumentation

**Fig. 6:**
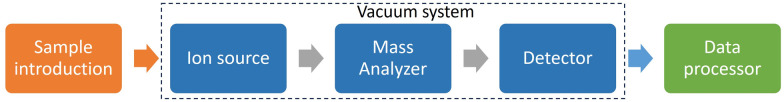
Schematic of the main steps commonly used in lab-based mass spectrometry analyses. There are many different types of mass spectrometers, but they all have three features in common. The first is some means by which atoms or molecules from the sample can be ionized. Neutral species cannot be steered by electric fields used in mass spectrometers, and thus it is necessary to produce ions. There are many different means by which this can be accomplished, and they are collectively referred to as ion sources. The second component of all mass spectrometers is the mass analyzer itself. There are several different means by which the *m*/*z* ratio of ions can be measured. Time-of-flight (ToF), orbitraps and quadrupole mass analyzers are commonly used, each with its own set of strengths and limitations. The final component common to all mass spectrometer systems is a detector for detecting or counting the number of ions with a specific *m*/*z* value. A final factor that needs consideration is how to couple the ion source to the sample to produce the ions for measurement, especially because all mass spectrometers must be operated under vacuum. In some cases, the sample will also be housed under vacuum, while in other cases the sample will be at atmospheric pressure and some may incorporate some other form of separation technology prior to introduction into the ionization chamber.

Even though we may not notice, mass spectrometry analysis is embedded in diverse aspects of day-to-day life. Bodily fluids drawn for medical testing may be analyzed by MS. The doped semiconductors that form the basis of all our electronic devices use MS as part of the quality control process. The use of MS helps identifying potential explosives before they make it on board an airplane and can also assist in finding toxins in our food supply. Mass spectrometry is a form of chemical analysis used to measure the mass-to-charge ratio (*m*/*z*) of atoms and/or molecules in a sample and it can be accomplished using a larger variety of platforms (Fig. 6). It is also capable of distinguishing between different isotopes of the same element. Depending upon the type of mass spectrometer, these measurements can often be used to determine the exact molecular weight of the sample components and to identify unknown compounds.

### 6.2 Miniature mass spectrometers

**Fig. 7:**
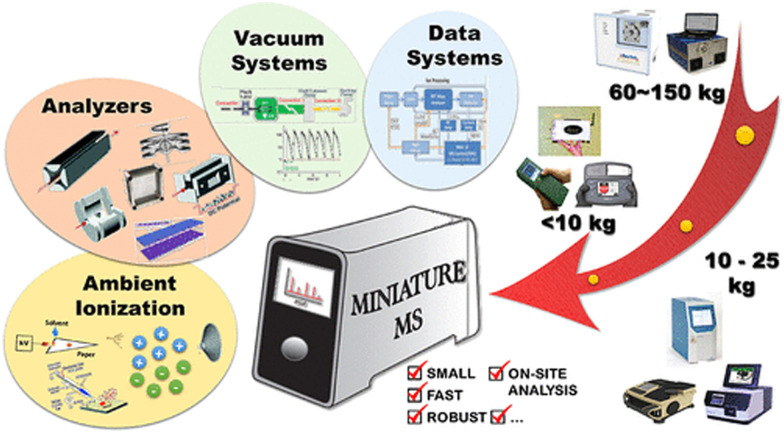
Graphical abstract of a review about miniature and fieldable mass spectrometers adapted from ref. 217. Some miniature mass spectrometer analyzers are currently providing unit resolution up to an upper mass of *m*/*z* = 1000. These systems have been continuously shrinking in size with adoption of microelectromechanical system technologies. These mass analyzers can provide compound identification/confirmation and quantitation limits close to benchtop instruments for some warfare and environmental compounds operating on battery power for a few hours. Many ionization techniques are available, especially ambient methods, to tailor the analysis to a specific application, and the entire system is expected to be light and small. A long development period preceded these advances. The development began with the first hand-held mass analyzer reported in 1991, proceeding to the first arrays of quadrupoles, as well as serial and parallel ion trap arrays along with the first micro-ion trap arrays. The introduction of ambient ionization and the first discontinuous atmospheric pressure interface resulted in fully portable systems coupled to ambient ionization sources. Miniature mass spectrometers are expected to become a major tool in the analytical sciences, especially given the increasing interest in *in situ*, point-of-care, online, and on-site measurements. Adapted with copyright permission from ACS Publications.

The majority of research in the biomedical science field is carried out using high mass resolution instruments, such as orbitraps and QTOF mass analyzers. However, in the clinic, instrument footprinting, quantification needs and cost constraints necessitate the use of low-resolution devices and some of them are miniature mass spectrometers. Areas such as emergency response, military, and law enforcement, but also agriculture, home health care, archaeology, and pollution control are being benefited using portable mass spectrometers, which are having a fast pace of miniaturization (Fig. 7). Portability is a primary driver for miniaturization efforts, but factors such as ease of use are also strongly considered since users are expected to have little or no technical training in analytical chemistry. Although performance may be reduced from laboratory-scale instruments, the value of the instrumentation is only as good as the quality of information provided to allow critical decisions to be made.

A compilation of 35 lightweight mass spectrometers was reported by Snyder *et al.*^217^ The Mini lineage of instruments developed at Purdue University exemplifies well the concept of portability. The Mini 10^218^ and 11^219^ mass spectrometers are hand-held instruments of total weights 10 and 4 kg, respectively, which operate under low-power conditions (<70 W). They contain identical rectilinear ion traps that can isolate compounds from complex mixtures and perform structural analysis with unit resolution across a mass range of up to *m*/*z* = 700. While the Mini 10 used an internal electron impact source, the Mini 11 was designed with a discontinuous atmospheric pressure interface (DAPI) to take advantage of internal, external, and ambient sources. The Mini 12 is the newest member of Purdues lineup of miniature mass spectrometers and is designed for point-of-care analysis.^220^ Power consumption is similar for the Mini 10 and 11, 50 W, because the same trap is used. The Mini 12 has an integrated solvent pump with solvent containers, sample cassette, and novice user interface for direct analysis of blood and other biofluids. The Mini S is an alternative backpack configuration that has similar performance characteristics but is designed for in-field applications (*e.g.*, pesticides, narcotics, and explosives detection).^221^ The usual ion source is a low-temperature plasma probe that operates independent of geometry and is integrated into a 2 kg hand-held unit. This unit also contains the ion transfer capillary, DAPI valve, rectilinear ion trap, and detector. The electronics, battery, vacuum system, and plasma gas, the heaviest components, are in the 10 kg backpack unit. Two miniaturized mass spectrometry systems have recently been developed by the Beijing Institute of Technology. The first is a typical DAPI/rectilinear ion trap instrument that couples capillary electrophoresis (CE) with nanoelectrospray.^222^ The system was able to separate a peptide (MRFA) from doubly charged angiotensin II, which has the same nominal *m*/*z*. A second instrument used a continuous atmospheric pressure interface rather than DAPI.^223^ This was achieved by differential pumping, where the first low-pressure region (1–6.6 torr) was separated from ambient pressure by the ion transfer capillary and separated from the mass analyzer region (1–6.6 mTorr) by a small aperture. The vacuum system was standard: a diaphragm pump from Scroll Tech combined with a Pfeiffer HiPace 10 turbo pump. The instrument showed excellent reproducibility (RSD <7%), low ppm limits of detection, and unit resolution. Other trap-based configurations with external/ambient ionization are MassTechs MT Explorer 50, which has a 3D ion trap and can be interfaced with ESI, atmospheric pressure matrix-assisted laser desorption/ionization (AP MALDI), atmospheric pressure chemical ionization (APCI), and direct analysis in real time (DART) sources, and an instrument with a low-pressure dielectric barrier discharge ionization source coupled to a linear ion trap.^224^ In this instrument, a diaphragm pump is used to pump down the sample container to transfer vapors through a pinch valve into the ion source. An AC voltage is applied to a dielectric, resulting in ionization of analytes. A reflectron-TOF developed by Shanghai University, Guangzhou Hexin Analytical Instrument Co., Ltd. and the National University of Defense Technology of the Peoples Liberation Army of China uses dimethylsiloxane membrane introduction and single photon ionization (UV) for detection of volatile components in air. A second TOF, the Suitcase TOF, was developed by the Johns Hopkins Applied Physics Laboratory.^225^ This breakthrough instrument is pumped by a standard diaphragm/turbo pump combination. Matrix assisted laser desorption/ionization is used as the source. The mass analyzer, a reflectron TOF, was able to detect 4 pmol bovine serum albumin, a 66 kDa protein, and showed performance similar to a commercial TOF in terms of resolution and sensitivity. Gas/air sampling is a popular target for portable analysis. The Sam Yang Chemical Company of Korea has developed a palm portable trap-based mass spectrometer for chemical warfare agent (CWA) determination weighing only 1.48 kg but pumped only by an ion getter (and thus requiring frequent recharge).^226^ The IonCam from OI Analytical, an instrument no longer commercially available but in the process of being updated, is built using Mattauch–Herzog sector geometry with gas chromatographic (GC) separation, allowing for simultaneous detection of ions of a range of masses. Other instruments for sampling gaseous analytes include the M908 from 908 Devices, which operates at high pressures (>1 torr) with an ion trap array and which can perform continuous vapor analysis or solid/liquid analysis with thermal desorption swabs. The MS-200 from Kore Technologies, Ltd has a membrane inlet, TOF analyzer, and nonmechanical pumps. Another instrument, the HAPSITE (Inficon), allows the detection of volatile and semivolatile organic compounds, while the ruggedized GUARDION-7 and TRIDION-9 GC/MS instruments from Torion Technologies (PerkinElmer) use toroidal ion traps.^227,228^ Other instruments to mention are a GC-quadrupole ion trap from the California Institute of Technology and Thorleaf Research;^229^ and a double focusing (ExB) instrument from the University of Costa Rica and University of Minnesota.^230^ Field-based chemical analysis in harsh environments presents challenges for the operator and instrumentation alike. To address the detection needs in such environments, ruggedization is a key attribute of portable chemical detection systems. Electrical systems and low-level sensors have been designed and reported to allow measurement at variable temperature and pressure, under toxic/caustic conditions, and at high radiation and electromagnetic pulse levels.^231–233^ Complex modern instruments, such as mass spectrometer devices, have components that can be susceptible to variable operating conditions. In recent years, miniaturization of all the components of mass spectrometers, from pumps and power supplies to mass analyzers and ionization sources, is rapidly advancing. Also, ambient ionization methods that allow for the direct, rapid, and high-throughput analysis of samples under open air conditions with minimal sample preparation are proliferating. Ambient ionization was introduced by Graham Cooks in 2004, when desorption electrospray (DESI) was reported.^234^ Over 80 ambient ionization strategies for the direct analysis of samples have been reported,^235^ including paper spray,^236^ DART,^237^ and rapid evaporative ionization mass spectrometry (REIMS).^238^ This has resulted in lowering the power requirements and allowing battery operation for a variety of applications such as rapidly identifying poisoning of patients in ambulances or emergency rooms, monitoring transoceanic shipping containers, and ensuring safe food and water supplies throughout the world. Such a device could accompany medical staff assisting patients in regions of widespread pandemic, where both time and laboratory access become critical constraints. Burns *et al.*^239^ have assessed the use of a deployable, single quadrupole mass spectrometer for its ability to detect explosives from glass fiber swabs using two different ambient ionization techniques. Both secondary electrospray ionization and pressure chemical ionization were found to be able to detect the five explosives studied from swabs at levels suitable for trace screening. Recently, researchers from the Naval Air Warfare Center, Weapons Division (NAWCWD) developed a 3D printed cone that can be used as a collection device and an ambient ionization source in field environments. It has been applied for the detection of chemical warfare agent simulants, environmental chemicals (perfluoroalkyl and polyfluoroalkyl substances – PFAS), and synthetic cannabinoids.^240^ The detection and identification of chemical warfare agents (CWAs) is a paradigm of health risk assessment in the field. Current methods of detection include color-changing paper, vibrational spectroscopy (infrared and Raman), ion mobility spectrometry and mass spectrometry.^241–244^ The detection, identification and quantification of volatile CWAs are well established in laboratory analysis *via* gas chromatography mass spectrometry (GC-MS) and this technology has been adapted for portable on-site use.^217,244^

## 7 Conclusions

Substantial advances have recently been made in the application of mass spectrometry-based approaches for evaluation of human health. Our improved ability to detect compositional variations in biofluids will continue to benefit diagnostic approaches as well as the development and implementation of personalized therapeutic strategies. The increased utilization of multiple omics platforms often being complementarily employed necessitates the development and optimization of integrative frameworks to create synergistic data sets. The need for method and metabolite identification standardization, quality control, and preanalytical procedures is key to data quality and the translation of biomarkers into diagnostic tools. These factors have been recently reported in a number of publications.^245–248^ The generation of these integrative data sets from multiple omics platforms should improve our understanding of complex biological processes often induced following exposure and during disease progression. Although often utilized and an extremely valuable component in the elucidation of biological responses, metabolomics currently is only capable of identifying a fraction of the metabolites within a biological sample. The expansion of metabolite information would unveil new targets and diagnostic markers. Lastly, the production of new mass spectrometry instruments to be utilized in the field or in clinical settings should enable the ability to make data-driven decisions in real-time for healthcare providers. Critical to the everyday use of these tools is the development of instrumentation and software that allows for minimum training and expertise in mass spectrometry. These devices will fundamentally alter medicine and field applications through the ability of novice users from a variety of disciplines to apply this powerful and precise technology.

## Author contributions

All authors mentioned in this article contributed to different stages of this study. The study design, materials preparation, data collection, and analyses were performed by Christina R Ferreira. The first draft of the manuscript was written by Christina R Ferreira. All authors contributed to interpreting the results and provided critical feedback, commenting on previous versions of the manuscript. The final version of the manuscript, the figures and the main text, was written and revised by Christina R. Ferreira, Paulo Clairmont F. de Lima Gomes, Bruce R. Cooper and Jonathan H. Shannahan. All authors read and approved the final manuscript.

## Conflicts of interest

The authors declare that there are no conflicts of interest.

## Supplementary Material
